# The association between subjective socioeconomic status and depressive symptoms in women of reproductive age: the chain mediating effects of marital satisfaction and well-being

**DOI:** 10.3389/fpsyg.2025.1715488

**Published:** 2025-12-05

**Authors:** Ruiyang Han, Xiao Ding, Manzhou Yang, Wenjia Feng, Yan Wang, Weiqin Cai, Qianqian Gao, Yuanzhuo Han, Youli Hu, Anning Ma

**Affiliations:** 1 School of Public Health, Shandong Second Medical University, Weifang, China; 2 School of Management, Shandong Second Medical University, Weifang, China; 3 Collaborative Innovation Center for Major Social Risk Prediction and Governance, “Healthy Shandong”, Weifang, China; 4 Second People’s Hospital of Weifang, Weifang, China

**Keywords:** women of reproductive age, subjective socioeconomic status, marital satisfaction, well-being, depressive symptoms

## Abstract

**Objective:**

The underlying mechanism between subjective socioeconomic status (SSS) and depressive symptoms among women of reproductive age in China is not fully. This study aims to explore the mediating roles of marital satisfaction and well-being in the association between SSS and depressive symptoms.

**Methods:**

A total of 4,219 women of reproductive age were selected from the 2022 China Family Panel Studies. Data related to SSS, marital satisfaction, well-being, and depressive symptoms were extracted. Spearman rank regression and bootstrap methods were used to analyze the chain mediation effects of SSS, marital satisfaction, well-being, and depressive symptoms.

**Results:**

(1) SSS, marital satisfaction, well-being, and depressive symptoms were significantly correlated (*p* < 0.01). (2) SSS directly affected depressive symptoms (*β* = −0.1092, *p* < 0.001). (3) Marital satisfaction (*β* = −0.0873, *p* < 0.001) and well-being (*β* = −0.0867, *p* < 0.001) each played a mediating role in the effect of SSS on depressive symptoms. (4) Marital satisfaction and well-being played a chain mediating role in the association between SSS and depressive symptoms in women of reproductive age (*β* = −0.0703, *p* < 0.001).

**Conclusion:**

There is a chain mediation effect between SSS, marital satisfaction, well-being, and depressive symptoms in women of reproductive age. Improvement in SSS can enhance marital satisfaction, which in turn increases well-being, ultimately alleviating depressive symptoms in women of reproductive age.

## Introduction

Women of reproductive age typically refer to females between the ages of 15 and 49 who are in their childbearing years (Qian et al., 2024). According to the seventh national population census, the number of women of reproductive age in China is 320 million, accounting for 22.9% of the total population (National Bureau of Statistics, 2020). Women of reproductive age are not only important participants in the socio-economic development and core links in families, but they are also a vulnerable group when it comes to mental health issues. This is especially true for married women, who often face challenges from multiple aspects, such as work pressure, family responsibilities, and personal life (Kim et al., 2022; Maeda et al., 2019; Vincent et al., 2025). Their mental health not only affects their own quality of life but also has the potential to influence the next generation through intergenerational transmission. Therefore, focusing on the mental health of women of reproductive age has significant social implications.

Depression is a common mental disorder that can lead to low mood, impaired cognitive function, and even increase the risk of suicide (Han et al., 2024). Preventive and health-promoting actions at the individual level play a crucial role in reducing the prevalence of depression (Herrman et al., 2022). The World Health Organization (WHO) has highlighted that women are more susceptible to depression and anxiety compared to men, with depression being a leading contributor to the disease burden among women in both high-income and low- to middle-income countries (World Health Organization, 2018). A study by Dai et al. (2025) found that from 1990 to 2021, the global burden of depression among women of reproductive age showed a significant upward trend. A survey from rural Hubei province in China revealed that socioeconomic status, illness, and stress are negative factors influencing depressive symptoms among women of reproductive age (Cao et al., 2015). For women of reproductive age, depressive symptoms may also affect the physical and mental health of offspring, creating a negative intergenerational cycle (Motrico et al., 2023; Silverman et al., 2020).

Subjective socioeconomic status (SSS) refers to an individual’s subjective perception of their own social class and economic standing, and is considered to be more sensitive in reflecting an individual’s mental health status compared to socioeconomic status (SES) (Choi et al., 2015). A study from Iran showed that the mediating role of SSS between SES and General Health Questionnaire-28 (GHQ-28) scores accounted for over 80% (Nasirpour et al., 2024). Another study on East Asian countries (China, Japan, and Korea) indicated that SSS consistently predicted depression across these three countries (Zang and Bardo, 2019). In the Chinese context, Shu et al. (2024) investigated the relationship between subjective family status and depression in Chinese adolescents, finding a significant correlation between these two variables. Kwong et al. (2020) conducted a 4-year follow-up study on elderly individuals in Hong Kong and concluded that SSS is an independent predictor of long-term mood, highlighting that SSS can capture important aspects of social status that SES may not fully reflect. Tan et al. (2023) studied healthcare workers after emergency risk events and found that a higher SSS could reduce depression in healthcare workers. Collectively, studies from China and other countries suggest that low SSS increases the risk of depressive symptoms. When individuals perceive themselves to be in a lower social economic position, the relative deprivation they feel can give rise to emotions such as jealousy and depression (Li et al., 2025).

Marital satisfaction is the subjective evaluation of the quality of the marital relationship, encompassing various dimensions such as emotional support, conflict resolution, and role division (Nadolu et al., 2020). Marital satisfaction is considered a significant predictor of depressive symptoms, with marital discord being a risk factor for depression (Du et al., 2021; Yang L. et al., 2023). A harmonious marriage can buffer stress by providing positive support, thereby reducing the occurrence of depressive symptoms. Social capital and economic foundation are among the essential factors for maintaining marriage. Previous studies have pointed out that low SSS can lower marital satisfaction (Jia et al., 2025). Women of reproductive age, who bear more family responsibilities, are particularly vulnerable to fluctuations in socioeconomic status (Rashidi Fakari et al., 2022), such as career interruptions or income decline. These changes can reduce their influence in family decision making, leading to marital dissatisfaction and psychological distress.

Well-being is the subjective evaluation of an individual’s quality of life (Xiao and Huang, 2022), including assessments of both their objective environment and subjective emotional state. Research has indicated that high SSS can enhance well-being (Chen et al., 2022; Wang et al., 2023), and well-being itself is a related variable for predicting depressive symptoms (Tran et al., 2017; Grant et al., 2013). Marital satisfaction is also linked to well-being (Yang Y. et al., 2023), and a harmonious marital relationship can enhance emotional support, thereby improving well-being.

In the model of this study, social rank theory and relative deprivation theory serve as the theoretical foundation. Individuals form subjective perceptions of their socioeconomic status through comparisons with others. Downward comparisons bring relative satisfaction, while upward comparisons generate relative deprivation. The relative psychological feelings generated in this subjective environment are more likely to influence people’s attitudes and behaviors than the objective environment (Kraus and Keltner, 2013; Kraus et al., 2013; Smith et al., 2012). Based on these theories, this study introduces family stress theory. Reuben Hill defined family stress as the pressure resulting from a lack of resources when the family is in difficulty. The psychological pressure brought about by relative deprivation can erode the harmonious family structure, promoting the onset of depressive symptoms through marital dissatisfaction on both spouses’ part (Hill, 1951). Furthermore, marital satisfaction is part of an individual’s subjective experience. The Actor-Partner Interdependence Model (APIM) theory theoretically explains the association between marital satisfaction and well-being. In marriage, both spouses are not isolated individuals but interdependent subjects. A lack of well-being increases the risk of depression (Cook and Kenny, 2005).

The current research gap lies in the fact that existing studies have partially explored SSS, marital satisfaction, well-being, and depressive symptoms but have not integrated these four variables into one model for analysis, particularly focusing on the vulnerable group of women of reproductive age. Therefore, this study incorporates social rank theory, relative deprivation theory, family stress theory, and the APIM theory into a single model using chain mediation. It posits that depressive symptoms in women of reproductive age are not only influenced by social comparison but also related to family stress and positive psychological resources. By exploring the relationships among these four variables, this study provides insights at the social and family levels for future interventions in depressive symptoms among women of reproductive age.

In summary, although there have been studies on the association between SSS and depressive symptoms in various groups (Wu et al., 2023; Anna and Michael, 2023; Assari et al., 2018), research on how SSS affects depressive symptoms in women of reproductive age is relatively limited, especially studies related to chain mediation. Therefore, this study aims to explore the association between SSS and depressive symptoms in women of reproductive age, with a focus on the roles of marital satisfaction and well-being in this association.

The following hypotheses are proposed in this study:

Hypothesis 1: SSS negatively predicts depressive symptoms in women of reproductive age.

Hypothesis 2: Marital satisfaction mediates the association between SSS and depressive symptoms in women of reproductive age.

Hypothesis 3: Well-being mediates the association between SSS and depressive symptoms in women of reproductive age.

Hypothesis 4: Marital satisfaction and well-being jointly mediate the association between SSS and depressive symptoms in women of reproductive age through a chain mediation effect.

## Materials and methods

### Sample and data collection

**Figure 1:**
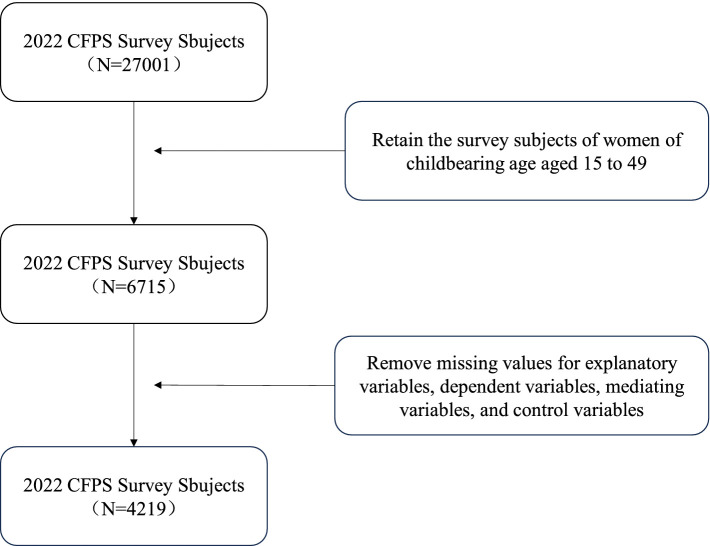
Variable screening process.

The data used in this study comes from the 2022 individual questionnaire of the China Family Panel Studies (CFPS), conducted by the Social Science Survey Center at Peking University. The CFPS uses a multi-stage stratified sampling approach to gather data at the individual, family, and community levels. It focuses on both the economic and non-economic welfare of Chinese residents, covering a wide range of research topics such as economic activities, educational outcomes, family dynamics, population migration, and health (Xie and Hu, 2015). Since this study involves marital satisfaction among women of reproductive age, and the legal marriage age for women in China is 20 years old, the study sample was limited to married women of reproductive age between 20 and 49 years. After excluding samples with missing key variables, a total of 4,219 complete individual samples were selected. The specific screening process is shown in Figure 1.

### Measure

#### Depressive symptoms

Depressive symptom severity was evaluated using the 8-item version of the Center for Epidemiologic Studies Depression Scale (CESD-8) (Van de Velde et al., 2009). The applicability of the CESD-8 has been confirmed in previous studies (Zhao et al., 2024). The CESD-8 consists of eight items, and participants are required to respond to the following eight questions: “I feel depressed,” “I feel strained doing anything,” “My sleep is not good,” “I feel happy,” “I feel lonely,” “I live a happy life,” “I feel sad,” and “I feel that life cannot continue.” Respondents answer these eight questions using four options: 1 = “Rarely or none of the time (less than 1 day),” 2 = “Some of the time (1–2 days),” 3 = “Most of the time (3–4 days),” and 4 = “All of the time (5–7 days).” These four options are scored from 1 to 4 points. The questions “I feel happy” and “I live a happy life” are positive emotion items, and these two items were reverse-scored (i.e., 1 = “Most of the time (5–7 days)” to 4 = “Almost none of the time (less than 1 day)”). The total score of the scale ranges from 8 to 32 points, with a higher score indicating more severe depressive symptoms in women of reproductive age. In this study, Cronbach’s *α* = 0.776.

#### SSS

Based on previous studies (Zhou et al., 2023; Yuan and Yueping, 2022), this study uses two questions from the CFPS questionnaire to assess the SSS of women of reproductive age: “How would you rate your social status in your locality?” and “How would you rate your income status in your locality?” Both questions use a 5-point scale (1 point representing very low to 5 points representing very high). The sum of the scores from these two questions is used as the composite score for SSS. The score range is from 2 to 10, with a higher score indicating a higher SSS. In this study, the mean score for the question “How would you rate your social status in your locality?” was 2.81 (standard deviation = 0.991), with a skewness of 0.082. The mean score for the question “How would you rate your income position in your locality?” was 2.83 (standard deviation = 1.000), with a skewness of 0.041. Cronbach’s *α* = 0.713.

#### Marital satisfaction

Based on previous research (Yang L. et al., 2023), this study measures marital satisfaction using three questions: “In general, how satisfied are you with your current marital life?,” “How satisfied are you with your spouse’s financial contribution to the family?,” and “How satisfied are you with your spouse’s contribution to the family in terms of household chores?.” The score range is 3 to 15 points, with a 5-point scale used for each question (1 representing very dissatisfied to 5 representing very satisfied). Higher scores indicate higher marital satisfaction. In this study, Cronbach’s *α* = 0.759.

#### Well-being

Based on previous studies (Sun et al., 2025), this study measures well-being using the question “How happy are you?” from the CFPS questionnaire, with scores ranging from 0 to 10, where higher scores indicate greater happiness among women of reproductive age.

#### Control variables

Based on previous research (Zhou et al., 2023; Sweeting and Hunt, 2015; Qiu et al., 2023; Chang et al., 2016), this study includes the following control variables: age, household registration (0 = “Rural,” 1 = “Urban”), education level (1 = “Illiterate/semiliterate,” 2 = “Primary school,” 3 = “Junior high school,” 4 = “Senior high school,” 5 = “College or above”), chronic illness (0 = “No,” 1 = “Yes”), physical exercise (0 = “No,” 1 = “Yes”), alcohol consumption (0 = “No,” 1 = “Yes”), smoking (0 = “No,” 1 = “Yes”), geographical distribution (1 = “Western China,” 2 = “Central China,” 3 = “Eastern China,” 4 = “Northeastern China”), caregiver during illness (1 = “Parents,” 2 = “Spouse,” 3 = “Children,” 4 = “Other family members,” 5 = “Non-family members,” 6 = “Did not get sick,” 7 = “No one/No care needed.”), and health change in the past year (1 = “Better,” 2 = “No change,” 3 = “Worse”).

### Statistical analysis

This study uses SPSS 27.0 and R 4.5.1 software for statistical analysis. The Harman single-factor test is used to check for common method bias. Categorical data are described using frequency (*N*) and percentage (*%*). The results of the Kolmogorov–Smirnov test indicated that SSS, marital satisfaction, well-being, and depressive symptoms do not follow a normal distribution (all *p* < 0.001). Therefore, non-normally distributed continuous data are described using the median (*P25, P75*). The Mann–Whitney U test and Kruskal–Wallis test are used for difference analysis. Spearman rank correlation analysis is used for correlation analysis. The chain mediation effect is tested using the PROCESS v4.0 macro program developed by Hayes (2017). After incorporating control variables, SSS was set as the independent variable (*X*), depressive symptoms as the dependent variable (*Y*), and marital satisfaction (*M1*) and well-being (*M2*) as mediating variables. The chain mediation effect is tested using the Bootstrap method with 5,000 iterations, which does not involve the population distribution or its parameters, making it suitable for non-normally distributed variables in this study. A chain mediation effect is considered statistically significant if the 95% confidence interval (CI) does not include 0. This study uses two methods for sensitivity analysis: causal mediation analysis proposed by Imai et al. (2010) and the method of adding control variables (Coleman et al., 2020; McNeilly et al., 2022), to assess the robustness of the chain mediation model. Sensitivity analysis was performed using the “mediation” package in R and SPSS PROCESS v4.0. The significance level is set at *α* = 0.05.

## Results

### Common method bias test

The data in this study are all based on self-reports from the participants, which may lead to common method bias. Therefore, the Harman single-factor test was used to conduct an unrotated principal component analysis on all 15 items related to depressive symptoms, SSS, marital satisfaction, and well-being. The results showed that there were four factors with eigenvalues greater than 1, and the variance explained by the first factor was 32.88% (below the critical threshold of 40%). This indicates that there is no significant common method bias in this study.

### Descriptive statistical analysis

A total of 4,219 women of reproductive age were included in this study. The median age was 36 (32, 43) years. The median SSS score was 6 (5, 6), the median CESD-8 score was 14 (11, 17), the median marital satisfaction score was 12 (10, 14), and the median well-being score was 8 (6, 9). Women of reproductive age in rural areas, illiterate/semi-illiterate, those with chronic illness, those who did not participate in physical exercise, those from the western region, those with no caregiver or no care needed, and those whose health has worsened in the past year had higher CESD-8 scores. No significant differences in depressive symptoms were found in relation to alcohol consumption or smoking (*p* > 0.05) (Table 1).

**Table 1:** Descriptive statistical analysis (*N* = 4,219).

Variable	*N* (%)	Depressive symptoms *M(P_25_, P_75_)*	*Z/H*	*p*	*M(P_25_, P_75_)*
Household registration			−4.824	<0.001	
Rural	1,802 (42.7)	14 (11, 17)			
Urban	2,417 (57.3)	13 (11, 16)			
Education level			90.940	<0.001	
Illiterate/semiliterate	329 (7.8)	16 (13, 19)			
Primary school	590 (14.0)	14 (12, 17)			
Junior high school	1,500 (35.6)	14 (11, 17)			
Senior high school	643 (15.2)	14 (11, 16)			
College or above	1,157 (27.4)	13 (11, 16)			
Chronic illness			−5.043	<0.001	
No	3,863 (91.6)	14 (11, 16)			
Yes	356 (8.4)	15 (12, 18)			
Physical exercise			−6.890	<0.001	
No	2,689 (63.7)	14 (11, 17)			
Yes	1,530 (36.3)	13 (11, 16)			
Alcohol consumption			−0.265	0.791	
No	4,129 (97.9)	14 (11, 17)			
Yes	90 (2.1)	14 (10.75, 17)			
Smoking			−0.708	0.479	
No	4,164 (98.7)	14 (11, 17)			
Yes	55 (1.3)	14 (11, 17)			
Geographical distribution			60.396	<0.001	
Western China	1,200 (28.4)	14 (12, 17)			
Central China	1,124 (26.6)	14 (11, 16)			
Eastern China	1,425 (33.8)	14 (11, 16)			
Northeastern China	470 (11.1)	13 (10, 16)			
Caregiver during illness			116.950	<0.001	
Parents	238 (5.6)	14 (11, 17)			
Spouse	1,897 (45.0)	14 (11, 16)			
Children	88 (2.1)	14 (11, 17)			
Other family members	31 (0.7)	14 (11, 17)			
Non-family members	39 (0.9)	16 (13, 17)			
Did not get sick	737 (17.5)	13 (10, 16)			
No one/no care needed	1,189 (28.2)	15 (12, 18)			
Health change in the past year			221.041	<0.001	
Better	408 (9.7)	13 (10, 16)			
No change	2,687 (63.7)	13 (11, 16)			
Worse	1,124 (26.6)	15 (13, 18)			
Age					36 (32, 43)
Subjective socioeconomic status					6 (5, 6)
Depressive symptoms					14 (11, 17)
Marital satisfaction					12 (10, 14)
Well-being					8 (6, 9)

### Spearman rank correlation analysis

Among women of reproductive age, SSS was negatively correlated with depressive symptoms (*p* < 0.01), marital satisfaction was negatively correlated with depressive symptoms (*p* < 0.01), and well-being was negatively correlated with depressive symptoms (*p* < 0.01). Additionally, SSS was positively correlated with both marital satisfaction and well-being (*p* < 0.01), and marital satisfaction was positively correlated with well-being (*p* < 0.01) (Table 2).

**Table 2:** Spearman rank correlation analysis.

Variable	Subjective socioeconomic status	Marital satisfaction	Well-being	Depressive symptoms
Subjective socioeconomic status	1			
Marital satisfaction	0.260^**^	1		
Well-being	0.270^**^	0.535^**^	1	
Depressive symptoms	−0.151^**^	−0.269^**^	−0.339^**^	1

^∗^Significant at the 0.05 level (two-tailed). ^∗∗^Significant at the 0.01 level (two-tailed).

### Analysis of chain mediation effects

Based on the results of the descriptive and correlation analyses, this study conducted a chain mediation analysis with SSS as the independent variable, depressive symptoms as the dependent variable, and marital satisfaction and well-being as mediating variables, controlling for household registration, education level, chronic illness, physical exercise, and geographical distribution. The results showed that, among women of reproductive age, SSS negatively predicted depressive symptoms (*β* = −0.1092, *p* < 0.001), supporting Hypothesis 1. After incorporating marital satisfaction and well-being into the model, it was found that SSS positively predicted both marital satisfaction (*β* = 0.3869, *p* < 0.001) and well-being (*β* = 0.1693, *p* < 0.001). Higher marital satisfaction was significantly correlated with higher well-being (*β* = 0.3546, *p* < 0.001) and fewer depressive symptoms (*β* = −0.2258, *p* < 0.001), and well-being negatively predicted depressive symptoms (*β* = −0.5121, *p* < 0.001) (Table 3).

**Table 3:** Regression analysis of subjective socioeconomic status, marital satisfaction, and well-being on depressive symptoms.

Variable	Model 1:marital satisfaction (M1)				Model 2:well-being (M2)				Model 3:depressive symptoms (Y)			
	*β*	*Se*	*t*	95% CI	*β*	*Se*	*t*	95% CI	*β*	*Se*	*t*	95% CI
Subjective socioeconomic status (X)	0.3869^***^	0.0233	16.5940	(0.3412,0.4326)	0.1693^***^	0.0145	11.6742	(0.1409,0.1977)	−0.1092^***^	0.0326	−3.3468	(−0.1732, −0.0452)
Marital satisfaction (M1)					0.3546^***^	0.0093	38.1889	(0.3364,0.3729)	−0.2258^***^	0.0239	−9.4588	(−0.2726, −0.1790)
Well-being (M2)									−0.5121^***^	0.0341	−15.0019	(−0.5791, −0.4452)
Age	−0.0104	0.0063	−1.6404	(−0.0228,0.0020)	−0.0129^***^	0.0038	−3.3760	(−0.0204, −0.0054)	−0.0384^***^	0.0085	−4.5364	(−0.0550, −0.0218)
Household registration	−0.2038	0.0888	−2.2963	(−0.3778, −0.0298)	0.1112	0.0535	2.0787	(0.0063,0.2162)	−0.0731	0.1186	−0.6166	(−0.3057,0.1594)
Education level	−0.1059^**^	0.0393	−2.6954	(−0.1829, −0.0289)	0.0772^**^	0.0237	3.2568	(0.0307,0.1236)	−0.4418^***^	0.0525	−8.4075	(−0.5448, −0.3388)
Chronic illness	−0.1845	0.1462	−1.2621	(−0.4710,0.1021)	−0.2265^*^	0.0881	−2.5715	(−0.3992, −0.0538)	0.8553^***^	0.1953	4.3797	(0.4724,1.2381)
Physical exercise	0.0537	0.0881	0.6100	(−0.1190,0.2264)	0.2030^***^	0.0531	3.8236	(0.0989,0.3070)	−0.4655	0.1178	−3.9523	(−0.6964, −0.2346)
Geographical distribution	0.1661***	0.0419	3.9650	(0.0840,0.2482)	0.1160^***^	0.0253	4.5856	(0.0664,0.1656)	−0.2498^***^	0.0562	−4.4482	(−0.3599, −0.1397)
Constant	10.0987^***^	0.3261	30.9676	(9.4594,10.7380)	2.0833^***^	0.2178	9.5672	(1.6564,2.5102)	24.7387^***^	0.4875	50.7419	(23.7829,25.6946)
*R*	0.2648^***^				0.5700^***^				0.4352^***^			
*R^2^*	0.0701				0.3249				0.1894			
*F*	45.3508				253.2647				109.2414			

^∗^Significant at the 0.05 level (two-tailed). ^∗∗^Significant at the 0.01 level (two-tailed). ^∗∗∗^Significant at the 0.001 level (two-tailed).

**Figure 2:**
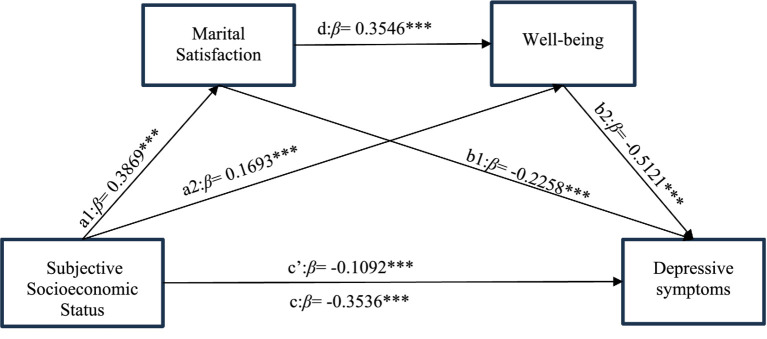
The chain mediation effect model of marital satisfaction and well-being in the association between subjective socioeconomic status and depressive symptoms. ^∗^Significant at the 0.05 level (two-tailed). ^∗∗^Significant at the 0.01 level (two-tailed). ^∗∗∗^Significant at the 0.001 level (two-tailed).

Using SPSS PROCESS macro Model 6 with control variables, the mediation effect of marital satisfaction and well-being in the association between SSS and depressive symptoms was tested. The results are shown in Table 4. The total effect was significant (95% CI: −0.4189, −0.2882), the direct effect was significant (95% CI: −0.1732, −0.0452), and the total indirect effect was significant (95% CI: −0.2825, −0.2083). Marital satisfaction had a significant mediating effect in the association between SSS and depressive symptoms in women of reproductive age (95% CI: −0.1116, −0.0653), supporting Hypothesis 2. Well-being also had a significant mediating effect in the association between SSS and depressive symptoms (95% CI: −0.1101, −0.0656), supporting Hypothesis 3. Additionally, marital satisfaction and well-being had a significant chain mediation effect in the association between SSS and depressive symptoms (95% CI: −0.0867, −0.0556), supporting Hypothesis 4. The chain mediation model of marital satisfaction and well-being in the association between SSS and depressive symptoms in women of reproductive age is shown in Figure 2.

**Table 4:** Significance test of mediation effects.

Path	Effect	Boot SE	Boot 95%CI	Percentage (%)
Total effect	−0.3536	0.0333	(−0.4189, −0.2882)	
Direct effect	−0.1092	0.0326	(−0.1732, −0.0452)	30.88%
Total mediation effect	−0.2443	0.0188	(−0.2825, −0.2083)	69.09%
Subjective socioeconomic status—Marital satisfaction—Depressive symptoms	−0.0873	0.0118	(−0.1116, −0.0653)	24.69%
Subjective socioeconomic status—Well-being—Depressive symptoms	−0.0867	0.0112	(−0.1101, −0.0656)	24.52%
Subjective socioeconomic status—Marital satisfaction—Well-being—Depressive symptoms	−0.0703	0.0079	(−0.0867, −0.0556)	19.88%

### Sensitivity analysis

**Figure 3:**
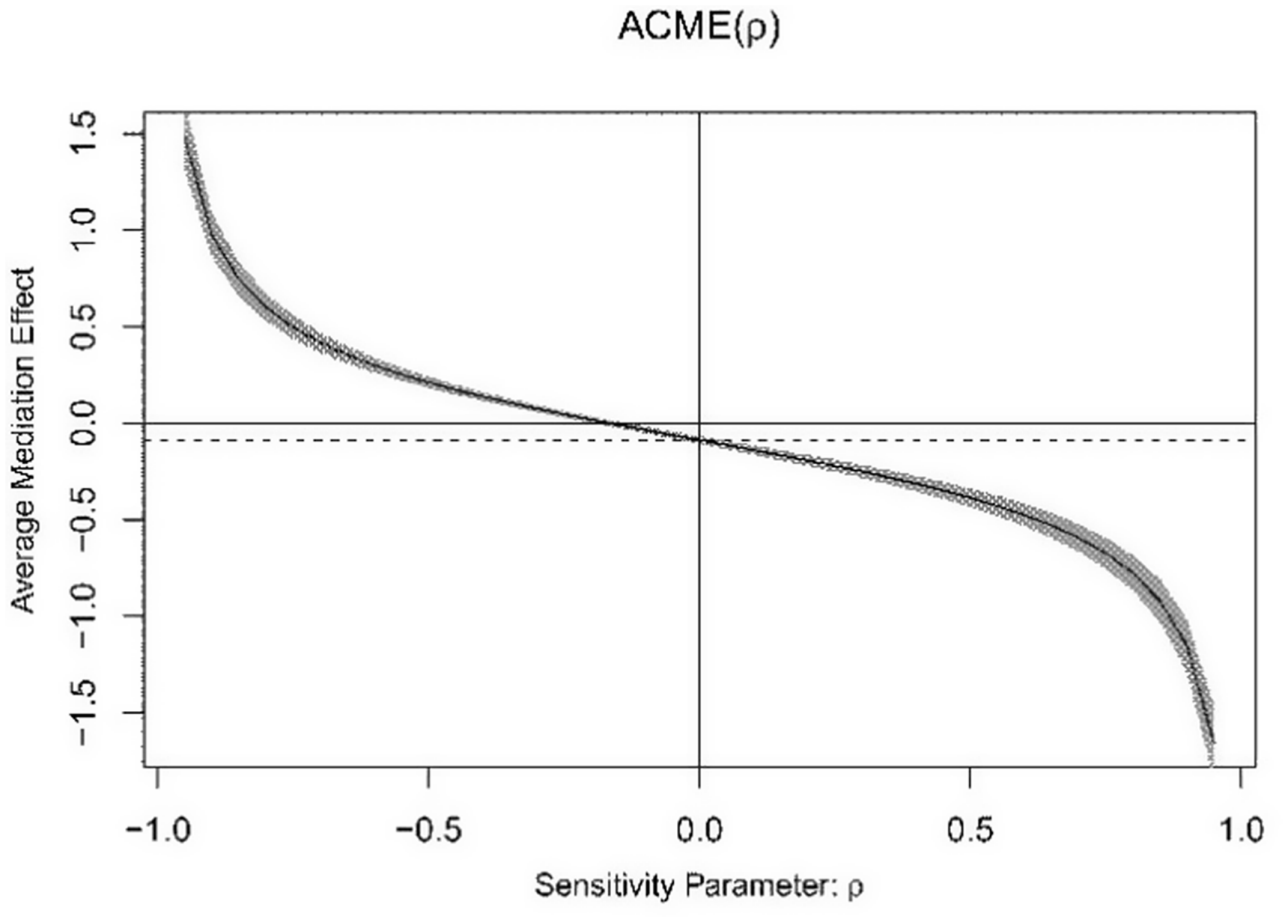
Average causal mediation analysis of Model 1.

**Figure 4:**
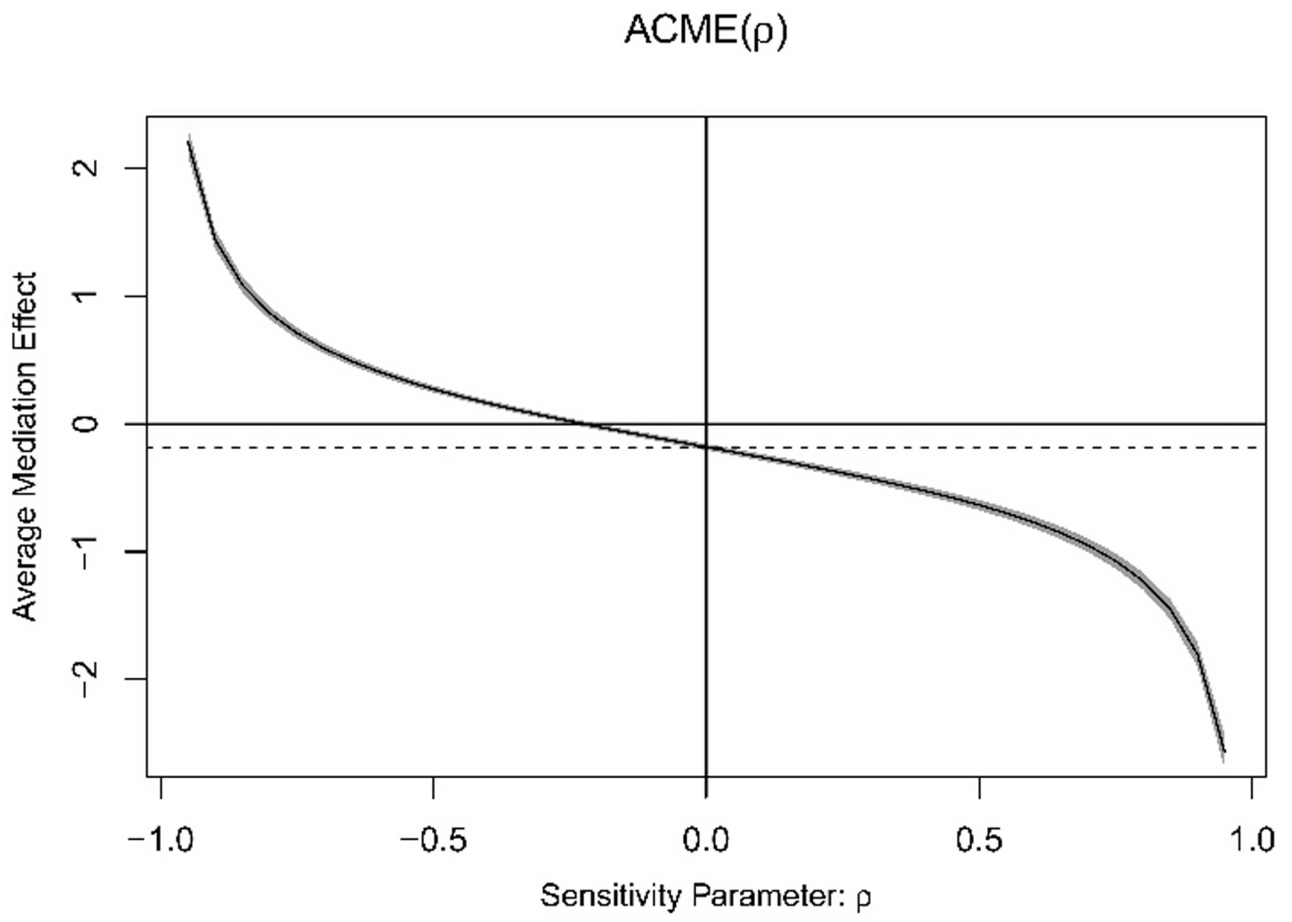
Average causal mediation analysis of Model 2.

The average causal mediation analysis was conducted separately for Model 1 and Model 2. The results showed that when the average causal mediation effect (ACME) of the model was 0, the rho value for Model 1 must be at least −0.15, and the rho value for Model 2 must be at least −0.25 (Figures 3, 4). By adding the caregiver during illness and health change in the past year as additional control variables into Model 3, the results indicated that the chain mediation effect still held (Table 5). The sensitivity analysis suggests that the mediation effect in this study is robust.

**Table 5:** The mediation effect values in the sensitivity analysis.

Path	Effect	Boot SE	Boot 95%CI
Total effect	−0.2954	0.0330	(−0.3601, −0.2307)
Direct effect	−0.0820	0.0323	(−0.1454, −0.0186)
Total mediation effect	−0.2134	0.0177	(−0.2496, −0.1792)
Subjective socioeconomic status—Marital satisfaction—Depressive symptoms	−0.0714	0.0106	(−0.0930, −0.0518)
Subjective socioeconomic status—Well-being—Depressive symptoms	−0.0818	0.0107	(−0.1040, −0.0617)
Subjective socioeconomic status—Marital satisfaction—Well-being—Depressive symptoms	−0.0602	0.0070	(−0.0749, −0.0472)

## Discussion

This study used data from the 2022 CFPS to conduct a cross-sectional analysis on women of reproductive age, examining SSS, marital satisfaction, well-being, and depressive symptoms. The results revealed that marital satisfaction and well-being served as a sequential mediator in the association between SSS and depressive symptoms in this group. The total mediation effect accounted for 69.09%, explaining most of the total effect. This reflects the significant role of the family system and individual positive emotions in alleviating depressive symptoms in women of reproductive age, providing a theoretical reference for multi-level approaches to ensuring the mental health of women of reproductive age.

### SSS influences depressive symptoms in women of reproductive age

The results of this study found that SSS was negatively correlated with depressive symptoms (*β* = −0.1092). The direct effect explained 30.88% of the variation of total effect, indicating that the higher the SSS, the lower the risk of depressive symptoms among women of reproductive age, which is consistent with previous studies (Hoebel et al., 2017; Assari et al., 2018; Callan et al., 2015). Both the Social Rank Theory of Depression (Wetherall et al., 2018) and the Relative Deprivation Theory (Mummendey et al., 1999) support the idea that depression can arise through social comparison. According to the Social Rank Theory of Depression, when an individual perceives themselves to be in a lower rank or disadvantaged position within a group, the frustration caused by this status gap significantly lowers their psychological evaluation of their own socioeconomic status, leading to depression and other mental health issues (Carraturo et al., 2023; Xiong and Hu, 2022). The Relative Deprivation Theory also suggests that when individuals compare themselves to social reference groups and perceive a gap, they experience negative feelings of deprivation regarding their resources or rights, leading to self-deprecation, inferiority, and other negative psychological states (Cundiff and Matthews, 2017; Yang et al., 2021).

Combining the aforementioned theories with the specific circumstances of women of reproductive age, it can be observed that women in this group not only engage in horizontal comparisons with colleagues and friends around them but also experience vertical self-comparisons. Career interruptions caused by childbearing force women to choose between career development and family caregiving (Zhang L. et al., 2024). The results of the Fourth China Women’s Social Status Survey show that only 2.7% of children under the age of 3 are primarily cared for by childcare institutions during the day, while 63.7% are cared for by their mothers (The Fourth Chinese Women’s Social Status Survey Office, 2022), reflecting the heavy caregiving burden on women of reproductive age, making it difficult to balance career development. This disparity caused by self-comparison, where income and status decline, may lead to a sense of relative deprivation, weaken their perception of their own socioeconomic status, and result in depression.

### Marital satisfaction mediates the association between SSS and depressive symptoms

Marital satisfaction mediates the association between SSS and depressive symptoms, accounting for 24.69% of the mediation effect. The higher the SSS of women of reproductive age (*β* = 0.3869), the higher their satisfaction with their marital association (*β* = −0.2258). These findings are consistent with previous research results (Leng et al., 2021; Moore et al., 2017). Higher marital satisfaction, in turn, reduces the risk of depressive symptoms in women of reproductive age. From the perspective of Family Stress Theory, changes in status perception due to family economic difficulties and lack of social resources can increase tension in marital relationships, leading to escalating family conflicts (Papp et al., 2009). Couples with low SSS are more likely to experience dissatisfaction with each other and less mutual understanding, thereby reducing marital quality.

However, marriage is a key source of emotional support, and a decrease in marital satisfaction removes a critical pathway for buffering stress. In the Marital Discord Model of Depression (MDMD), marital discord is considered a significant risk factor for depressive symptoms (Beach et al., 1990). Research by Whisman (2001) suggests that women are more likely to experience depressive symptoms than men as a result of reduced marital satisfaction. Similarly, Yang L. et al. (2023) using the Actor-Partner Interdependence Moderation Model (APIMoM), found a strong negative correlation between individuals’ marital satisfaction and both their own and their spouse’s depressive symptoms, with wives being more susceptible to depressive symptoms than husbands. Thus, making significant contributions to family finances and maintaining a harmonious marital relationship are important ways to effectively reduce depressive symptoms in women of reproductive age. Maintaining a good marital relationship serves as a protective factor for women of reproductive age in combating depressive symptoms.

### Well-being mediates the association between SSS and depressive symptoms

Well-being mediates the association between SSS and depressive symptoms, accounting for 24.52% of the mediation effect. Women of reproductive age with higher SSS have higher well-being (*β* = 0.1693), and higher well-being is associated with a lower risk of depressive symptoms (*β* = −0.5121). These findings are consistent with previous research (Grant et al., 2013; Huang et al., 2017). Positive psychology advocates for discovering one’s positive factors, and positive emotions are considered the best “weapon” to combat psychological disorders (Seligman, 2008). A meta-analysis by Tan et al. (2020) indicated that SSS is more strongly correlated with well-being than objective socioeconomic status. Curhan et al. (2014) analyzed the association between SSS and SES and mental health in the United States and Japan, finding that both could predict well-being. For women of reproductive age, those with higher SSS may have better access to material resources such as healthcare and education. Furthermore, high SSS, as a product of higher social rank, enhances self-worth and a sense of social respect, thereby increasing well-being. In contrast, low SSS may increase stress due to perceptions of insufficient economic and social support, which could diminish well-being.

In this study, a decrease in well-being was found to increase the risk of depressive symptoms. A study of Mexican women also highlighted the association between well-being and depressive symptoms, noting that lower well-being scores could evolve into a risk factor for severe depression (Hernández-Muñoz et al., 2022). Women of reproductive age are in a unique phase of life, where estrogen influences cognitive, behavioral, and emotional processes by regulating neurotransmitter systems (Colzato and Hommel, 2014), making them more susceptible to emotional fluctuations. Additionally, during this period, women face various social issues and conflicts, including parenting pressure, elder care, and housing loans, among others. The accumulation of negative emotions over time negatively affects well-being, ultimately leading to the emergence of depressive symptoms.

### Marital satisfaction and well-being serve as a chain mediating effect between SSS and depressive symptoms

Enhancing SSS can boost marital satisfaction, which in turn improves well-being and helps alleviate depressive symptoms in women of reproductive age, with the mediation effect accounting for 19.88%. This study also identified a decline in marital satisfaction as a predictor of lower well-being (*β* = 0.3546), aligning with the findings of Cook and Kenny (2005) based on the Actor-Partner Independence Model. In marital relationships, when one partner experiences dissatisfaction, they may attribute the blame to their spouse, thereby causing negative psychological effects on the other person. Conversely, individuals in happy marriages are more likely to provide emotional support and positive reinforcement to their partners, and such positive interactions help to increase the well-being of both spouses (Thomas et al., 2017). The positive emotional support derived from enhanced well-being can assist women of reproductive age in better coping with the pressures and challenges of life, reducing the occurrence of depressive symptoms.

This pathway indicates that the association between SSS and depressive symptoms is not a single, direct process, but also involves the interaction between family dynamics and individual subjective psychological experiences. Women with higher SSS may improve marital quality through economic resources and social support, which in turn enhances their well-being. Higher well-being, in turn, may help them face marital challenges with a more positive attitude, creating a virtuous cycle. In conclusion, marital satisfaction and well-being play independent mediating roles between SSS and depressive symptoms in women of reproductive age, and they also exhibit a chain mediation effect.

### Implications

To improve the mental health of women of reproductive age, this study proposes several recommendations based on the research findings. Firstly, there is a need to promote structural support policies aimed at enhancing women’s perception of their SSS. Efforts should focus on building a birth-friendly society that alleviates the economic and caregiving burdens faced by women of reproductive age. This can be achieved through the implementation of national policies such as paid parental leave (Mensah and Adjei, 2020), childcare subsidy (Yang, 2025), and personal income tax deductions (Yang and Zhang, 2023), as well as exploring universal childcare services (Horwood et al., 2021). These measures will help women balance work and family responsibilities, reduce the motherhood penalty (Yu and Liang, 2022), enhance income security, and improve career continuity, ultimately strengthening their SSS perception.

Secondly, it is crucial to strengthen family support and incorporate marital relationships into grassroots public services. Marital and family development should be integrated with community governance, and community grid-based services can be leveraged to provide public services like marriage communication skills training (Nejatian et al., 2021) and family conflict mediation. These initiatives will help women of reproductive age and their spouses build healthy interaction patterns, improve marital satisfaction (Alipour et al., 2020), and strengthen the emotional support provided by marriage.

Thirdly, targeted psychological interventions should be implemented with a focus on vulnerable groups. More focus should be directed towards the mental health of women of reproductive age at high risk of depression, especially in rural and western areas of China (Wang et al., 2024). These regions have lower levels of socioeconomic development and are more susceptible to negative events such as life pressures and traditional cultural biases, leading to pessimistic emotions. For these groups, a variety of mental health support strategies should be implemented, including regular monitoring of their well-being, the dissemination of mental health information, and the use of digital technology to offer remote psychological counseling (Zhen et al., 2024). This would overcome geographic limitations, improve mental health levels, benefit women in remote areas, and enhance their well-being.

Due to the limitations of the questionnaire, several potential variables that could be relevant to the model were not included. Different personality traits are often related to social and environmental factors, such as one’s social status and marital status (Zhang N. et al., 2024). Personality traits are also important factors influencing depressive symptoms. Previous research has pointed out that, according to the vulnerability-stress model, individuals possessing vulnerability or susceptibility traits are more susceptible to psychological issues like anxiety, depression, and loneliness when confronted with stressful events (He et al., 2025). A history of mental health issues or a family history of psychiatric disorders can influence current depressive symptoms through a vicious cycle, which may affect an individual’s perception of their socioeconomic status (Brandon et al., 2012; Bavle et al., 2016). Additionally, based on the stress process theory, stress can directly or indirectly lead to depressive symptoms. Exposure to chronic stress conditions may continuously cause negative emotions, which in turn can result in an increase in the level of depressive symptoms (Wickrama et al., 2017).

## Limitations

It is also important to recognize the limitations of this study. Firstly, the cross-sectional design means that the results primarily reveal the associations between variables and the potential mediating effects, but cannot establish strict causal relationships. Future research could further confirm these findings through longitudinal analysis. Secondly, well-being was measured using a single item, which, although common in large social surveys, does not capture the multidimensional nature of the construct of well-being. Additionally, the measurements of SSS and marital satisfaction were relatively brief. While they demonstrated acceptable internal consistency in this study, compared to more established and comprehensive scales, their content and construct validity may be limited. Thirdly, all variables in this study were obtained through self-reported data from the respondents, which may introduce recall bias and reporting bias. Fourthly, although multiple variables were controlled in this study, potential confounding factors such as personality traits, mental health history, and stress were not included, which may affect the internal validity of the results. Fifthly, the sample in this study only included married women of reproductive age, which allowed for a focus on the role of marital satisfaction but also means the conclusions may not be generalizable to all women of reproductive age. Future research could compare groups with different marital statuses to more comprehensively analyze the impact of subjective socioeconomic status on the mental health of women of reproductive age.

## Conclusion

The research results indicate that the level of SSS affects depressive symptoms in women of reproductive age. Furthermore, this study found that the intervention effect of SSS on depressive symptoms in women of reproductive age is mediated through a sequential pathway involving marital satisfaction and well-being. Specifically, higher SSS enhances marital satisfaction, which in turn increases well-being, ultimately alleviating depressive symptoms. Policies should be implemented to enhance SSS from multiple perspectives, as this is crucial for promoting the construction of a birth-friendly society.

## Data Availability

Publicly available datasets were analyzed in this study. This data can be found here: https://www.isss.pku.edu.cn/cfps/.
